# The Obstetric Catechism

**Published:** 1853-05

**Authors:** 


*The Obstetric Catechism; containing two thousand three hundred and forty-seven questions and answers on Obstetrics proper.* By JOSEPH WARRINGTON, M. D. One hundred and fifty illustrations. Philadelphia: Edmond Barrington and Geo. D. Haswell. 1853. 12mo., pp. 445.

The author of this work disclaims for it any title to the character of a *text* book. He calls it a *test* book. "It is," he says, "your catechist or inquisitor, not to tell you any thing new, but enable you to determine what you do, or what you do not already know." Used for this end such books are undoubtedly convenient and useful, and we have no doubt that this work is well adapted to fulfill the object for which it was designed. The reader's attention cannot but be arrested by the title page. There are 2347 interrogatories to be answered before the medical student is master of all the important facts pertaining to obstetrics! The practitioner may gauge his knowledge of this branch of medicine by ascertaining how many of these questions he is already prepared to answer; and in prosecuting this inquiry he may possibly discover that on some points his stock of information is defective. If not, the satisfaction of thus proving the amplitude of his knowledge will sufficiently repay the labor of perusal.

As respects anæsthesia in labor, Dr. W. is a conservatist, conforming to the sentiment on the subject which, it is generally understood, prevails at Philadelphia. The flippant manner in which he disposes of the matter in the preface, is unworthy of the author. We quote: "I have thoughtfully refrained from alluding to the subject of anæsthesia in obstetric practice, having not much to say from my own experience in its use, and after stating my strong objection to making women transiently *drunk*, whenever any substitute may be successfully available, I have still preferred not to attempt in the text to prejudice the mind of the student against any preceptorial or professional bias he may have received."

